# Cross-Platform Comparison of Microarray-Based Multiple-Class Prediction

**DOI:** 10.1371/journal.pone.0016067

**Published:** 2011-01-11

**Authors:** Xiaohui Fan, Li Shao, Hong Fang, Weida Tong, Yiyu Cheng

**Affiliations:** 1 Pharmaceutical Informatics Institute, College of Pharmaceutical Sciences, Zhejiang University, Hangzhou, China; 2 Z-Tech Corporation, an ICF International Company at NCTR/FDA, Jefferson, Arizona, United States of America; 3 National Center for Toxicological Research (NCTR), US Food and Drug Administration, Jefferson, Arizona, United States of America; University of Minnesota, United States of America

## Abstract

High-throughput microarray technology has been widely applied in biological and medical decision-making research during the past decade. However, the diversity of platforms has made it a challenge to re-use and/or integrate datasets generated in different experiments or labs for constructing array-based diagnostic models. Using large toxicogenomics datasets generated using both Affymetrix and Agilent microarray platforms, we carried out a benchmark evaluation of cross-platform consistency in multiple-class prediction using three widely-used machine learning algorithms. After an initial assessment of model performance on different platforms, we evaluated whether predictive signature features selected in one platform could be directly used to train a model in the other platform and whether predictive models trained using data from one platform could predict datasets profiled using the other platform with comparable performance. Our results established that it is possible to successfully apply multiple-class prediction models across different commercial microarray platforms, offering a number of important benefits such as accelerating the possible translation of biomarkers identified with microarrays to clinically-validated assays. However, this investigation focuses on a technical platform comparison and is actually only the beginning of exploring cross-platform consistency. Further studies are needed to confirm the feasibility of microarray-based cross-platform prediction, especially using independent datasets.

## Introduction

Microarrays, as efficient tools to simultaneously monitor the expression of tens of thousands of genes, have been widely applied in both mechanistic and decision-making research during the past decade [1]–[4]. The large number of commercially available microarray platforms has expanded the use of the technology and made it more widely available to different laboratories. However, left unresolved is the issue of whether inter-platform differences may conceal or confound biologically significant information with respect to potential biomarkers and prediction models. Thus, the concern that one needs to stay within a particularly microarray platform manufacturer slows down the identification and qualification of genomic biomarkers [5].

The extent to which different microarray technologies influence the identification of differential gene expression has been addressed by a large number of studies and is the subject of a review paper [6]. Despite the conflicting information given by a handful of early published studies where both concordance[7]–[9] and discordance[10]–[12] between technologies was demonstrated, the maturation of microarray technology and data analysis methods has led to improved cross-platform correlations[6], [13]. Moreover, the first phase of FDA-led Microarray Quality Control project (MAQC-Ι) has further confirmed the reproducibility of the identification of differentially expressed genes across different platforms [5], [14]–[16]. These studies suggest that similar results should be expected regardless of microarray platform if appropriate experimental and analysis protocols are applied, meaning that mechanistic research can incorporate datasets from multiple sources without significant concern about platform-specific affects.

The clinical use of array-based diagnostics is relatively late in coming; this is partially due to the demand of a substantial number of patient samples to be used for training, since estimates of a predictor's error rate during model construction are more prone to be biased for small datasets[17]. Therefore, an attractive approach would be the re-use of relevant pre-existing sets of expression profiles as training data. Although researchers have demonstrated that reciprocal validation can be achieved using different patient cohorts and microarray platforms[18], few benchmark analyses have been carried out until recently to confirm the feasibility of re-using datasets obtained from different platforms for diagnostic models. Based on the toxicogenomics datasets generated in phase II of the MAQC project using both Rat Genome 230 2.0 Array (Affymetrix platform) and Rat Oligo 2-color G4130A Array (Agilent platform) on the same tissue samples, our recent study[19] evaluated and confirmed that high cross-platform concordance of predictive signature genes and classifiers can be achieved for binary classification. However, in reality, decision-making is not always binary. For example, subtype identification in disease diagnosis[20], [21], toxicant discrimination[22] and the stratification of toxicity severity in drug risk/safety assessment[23] can, in most cases, only be achieved using multiple-class prediction. Thus, the consistency of microarray platforms with regard to multiple-class prediction discussed in this study is also of importance to the future success of microarray-based predictive models in clinical application and safety evaluation.

The primary issue we addressed is the comparability of models constructed from different platforms. We then further evaluated cross-platform consistency with regard to whether predictive signature features selected on one platform could be directly used to train a model on the other platform and whether predictive models trained using one platform could predict datasets from the other platform with comparable performance. In this study, three commonly-used multi-class machine learning algorithms were applied: fuzzy k-nearest neighbors (FKNN)[24], [25], linear discriminant analysis (LDA)[26] and support vector machine (SVM)[27]. The results provide a baseline confirmation of the cross-platform consistency of multiple-class prediction.

## Materials and Methods

### Datasets

The same datasets and the way in which they were divided into training and test sets have been previously described [19]. All data is MIAME compliant and the raw data are available through GEO (series accession number: GSE16716) and ArrayTrack (http://www.fda.gov/nctr/science/centers/toxicoinformatics/ArrayTrack/). Rather than a binary score, the outcome variable selected was the RHI (Response to Hepatocellular Injury) score, which ranges from 0 to 2 that are associated with the severity of chemically-induced hepatotoxicity [23]. Briefly, the toxicogenomics datasets for Affymetrix Rat Genome 230 2.0 Array with 31,099 probe sets (AFX) and Agilent Rat Oligo 2-color G4130A Array with 22,075 probes (AGL) were profiled from the same set of 418 samples (RNA isolated from the liver from each of the 318 treated and 100 control rats), resulting in 418 and 318 arrays, respectively. For hybridizations performed on the Agilent platform, each of the 318 treated samples was labeled and hybridized against a pooled RNA sample generated from the control samples.

A prerequisite for platform comparison is that all datasets are represented by a common set of probes. Three different approaches were used to identify probes associated with the same transcript: SeqMap, RefSeq, and Unigene, resulting in 4860, 6312, and 9954 common transcripts[19], respectively. SeqMap is a sequence-based approach to identifying common probes generated, and was also used in the MAQC-Ι project [16]. RefSeq is a less restrictive method of matching Agilent probes with Affymetrix probes based on the RefSeq database, while Unigene is the least stringent approach for identifying matching probes across platforms using the Unigene database.

Due to the technological difference in experimental design between Affymetrix (intensity) and Agilent 2-color (ratio) platforms, three analysis configurations (ACs 1-3, illustrated in **Figure S1**) were designed to ensure that both datasets matched in comparison. AC 1 utilized the original datasets (i.e., AFX intensity vs. AGL ratio), while AFX datasets in AC 2 were converted to ratio and compared with the AGL ratio data, and AGL datasets in AC3 were converted to intensity and compared with the AFX intensity data. Briefly, the Affymetrix ratio data was calculated using its intensity data in a way similar to Agilent platform, i.e., treated samples were compared to an average of the corresponding samples. In AC3, the intensity data in AGL is the average value of Cy3 and Cy5 corresponding only to the treated samples. Note that the 318 arrays profiled from the same samples using both platforms were used in AC 2 and AC 3, while AFX in AC 1 retained the original 418 arrays. Combined with the three classification algorithms (FKNN, LDA, SVM), a total of 27 comparisons were carried out, corresponding to 3 ACs, each with 3 classifiers, and each classifier having 3 probe-mapping methods. Detailed information on the datasets, probe mapping procedures, and ACs has been published previously [19], [28], [29].

### Study design

**Figure 1:**
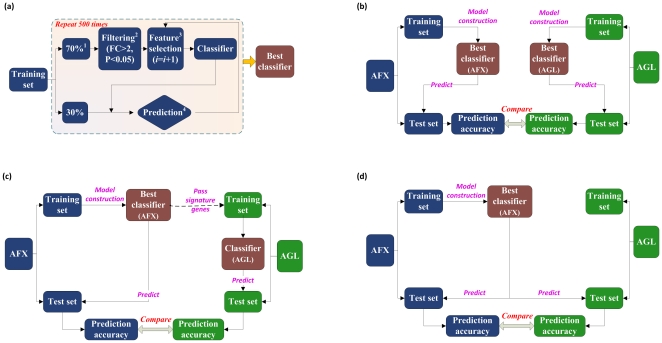
Detailed information on the study design. (a) Approach to development of the *best classifier*. (b) Assessment of performance of the *best classifiers* derived from different platforms. (c) Transferability of signature genes, i.e., whether predictive signature features selected in one platform could be directly used to train a model in the other platform. (d) Transferability of classifiers, i.e., whether predictive models trained using data from one platform could predict datasets profiled using the other platform with comparable performance.

Detailed information for the study design is illustrated in **Figure 1**; additional information about model construction procedures is available in **Supplementary Methods**. Both AFX and AGL datasets were divided into the predefined training and test sets. The analysis protocol starts with the construction of the *best classifier* using either the AFX or AGL training set **(** **Figure 1(a)** **)** and ends by using a *best classifier* to predict the test sets of both platforms. Corresponding to different destinations, three designs **(** **Figure 1(b–d)** **)** were utilized in this study.

To evaluate the performance of models constructed using different platforms, a *best classifier* was developed independently for both the AFX and AGL training data and then used to predict the corresponding test set. This procedure was repeated 500 times, resulting in 500 sets of predictions[1], [30]. The performance of models was then compared with respect to that of the overall samples and those in each subclass.

Next, signature genes selected in the *best classifier* on the training set of one platform (e.g., AFX) were transferred to the training set of the other platform (e.g., AGL) to train another classifier. This procedure was repeated 500 times, and the overall prediction accuracy as well as the prediction accuracy for each subclass was calculated and recorded.

Lastly, in order to evaluate whether classifiers developed from one platform could perform well on the other platform, the whole classifier (i.e., the *best classifier*) developed on the training set of one platform (e.g., AFX) was transferred to predict the test set of the other platform (e.g., AGL). The obtained prediction performance of test sets from both platforms for the 500 repetitions of the procedure were recorded and compared.

### T-index

The T-index score proposed in our previous study[19] was also used to evaluate the comparability of model performance metrics (e.g., accuracy) obtained from the two platforms. The T-index is defined as 
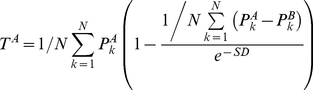
(1)where *T^A^* indicates the comparability degree, *N* is the number of iterations (*N* = 500), 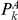 and 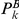 represents the prediction accuracies for the test sets of platforms *A* and *B* obtained from 500 iterations, respectively, and *SD* is the standard deviation of (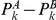). Note that T-index score ranges from 0 to 1, with a score smaller than 0.5 indicating the failure of transferability. In other words, a larger T-index score indicates better transferability.

## Results and Discussion

This is a benchmark analysis to evaluate the feasibility of re-using pre-existing datasets as training samples for multiple-class prediction models. We focused on the following three questions: First, do models constructed from different platforms have similar predictive performance both overall and for individual sub-classes? Second, can predictive signature genes selected from one platform be used to directly train a model on another platform? Lastly, can predictive classifiers trained on one platform perform well on data generated using another platform?

### Comparison of different microarray platforms

**Figure 2:**
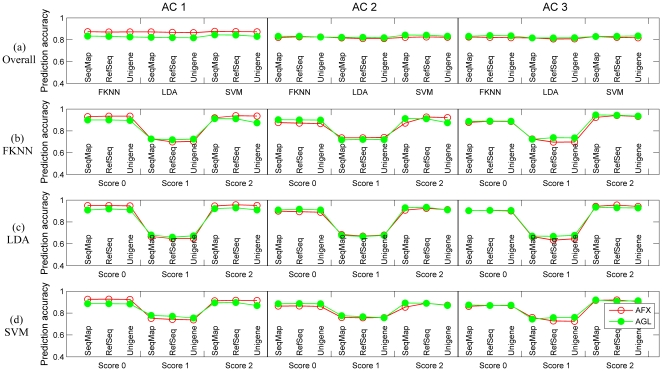
Comparison of different platforms. (a) Overall prediction accuracy for both test sets using models generated from each platform. Blue, yellow and brown bars represent ‘SeqMap’, ‘RefSeq’, and ‘Unigene’ for AFX, while corresponding circles faced green are for AGL. (b) Prediction accuracy for samples in each subclass using FKNN. (c) Prediction accuracy for samples in each subclass using LDA. (d) Prediction accuracy for samples in each subclass using SVM.

**Figure 2(a)** illustrates the overall prediction accuracy for models trained from both platforms on corresponding test sets using different combination of analysis configurations (ACs 1-3), probe matching protocols (SeqMap, RefSeq, Unigene), and classification algorithms (FKNN, LDA, SVM). No difference in predictive accuracy between the AFX and AGL datasets was observed for AC 2 and AC 3; however, AC 1 demonstrated slightly higher accuracy for AFX. Generally, probe matching protocols and classification algorithms showed no impact of overall predictive accuracy.

**Figure 2(b–d)** gives detailed illustrations of model performance for samples using FKNN, LDA, and SVM as classification algorithms, respectively. The nearly indistinguishable model performance of the AFX and AGL datasets in AC 2 and AC 3 further confirmed the comparability of different microarray platforms. Moreover, the consistently higher accuracy of AFX for samples with score 0 in AC 1 (**Figure 2(b–d)**) implies that the unexpected better performance of AFX in overall prediction accuracy (**Figure 2(a)**) might be attributable to the additional 100 control samples in AFX over AGL. Further evidence for this was given by the comparable performance of both platforms for overall samples and those in each subclass shown in **Figure S2**, where the 100 control datasets were removed and only the 318 treated samples were retained in the AFX dataset.

Generally, consistent model performance exists across different microarray platforms for multiple-class prediction, both for the complete set of samples and for those with different RHI scores, regardless of the ACs, probe-mapping methods, and classification algorithms. This strongly suggests that predictive models could be successfully developed using different microarray platforms as long as classifiers with the best performance could be constructed for each platform.

### Transferability of predictive signature genes

**Figure 3:**
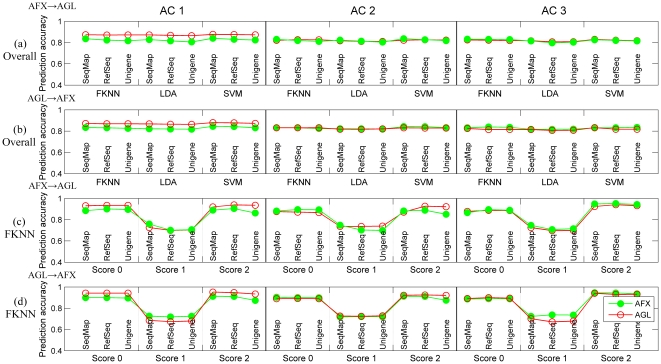
Transferability of predictive signature genes. (a) Overall prediction accuracy for both test sets using signature genes selected from AFX (AFX to AGL). (b) Overall prediction accuracy for both test sets using signature genes selected from AGL (AGL to AFX). In (a) and (b), blue, yellow and brown bars represent ‘SeqMap’, ‘RefSeq’, and ‘Unigene’ for AFX, while corresponding circles faced green are for AGL. (c) Prediction accuracy for samples in each subclass using FKNN in the transfer of AFX to AGL. (d) Prediction accuracy for samples in each subclass using FKNN in the transfer of AGL to AFX.

**Figure 3(a–b)** delineates the overall prediction accuracy for both test sets when signature genes selected from one platform were transferred to train a model in the other platform. Corresponding results for samples in each subclass using different classification algorithms are illustrated in **Figure 3(c–d)** **and Figure S3**. **Figure 3a** shows very similar performance in AC 2 and AC 3 for a model trained and tested on AFX data and a model using the same predictive features trained and tested on AGL data. Likewise, **Figure 3b** shows similar performance in ACs 2 and 3 for a model trained and tested on AGL data and a model using the same predictive features trained and tested on AFX data. This conclusion is further supported by corresponding T-index scores higher than 0.8 for most cases shown in **Tables S1 and S2**. Note that the relatively lower T-index scores around 0.72 for samples with a score of 1 should be attributed to the apparently worse performance in predicting such samples as shown in **Figure 3(c–d)** rather than poor transferability. As to the consistently higher performance of AFX in AC 1 for overall samples and those with a score of 0, it might also be ascribed to the additional 100 controls samples that were pooled for the AGL-generated data. The overlap of model performance shown in **Figure S4** supports not only the interpretations mentioned above, but also the successful transfer of signature genes using different ACs and probe-mapping methods.

These results provide excellent evidence that predictive signature genes selected from one platform can be successfully transferred to train a predictive model in the other platform regardless of the types of analysis configurations, probe-mapping methods, and classification algorithms used, as long as the datasets are capable of producing informative-enough predictive models (i.e., intrinsic predictable). This has the potential to improve the diagnostic use of array-based predictive models by avoiding the additional work and complexity of selecting different predictive signature genes for each platform, and allowing the combination of smaller datasets from multiple platforms that are not large enough on their own to obtain a highly informative gene set.

### Transferability of predictive classifiers

**Figure 4:**
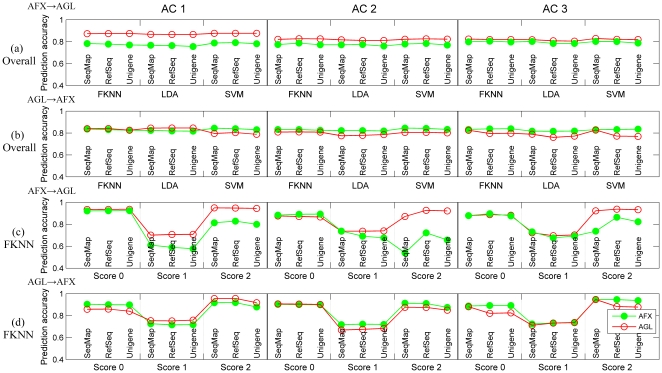
Transferability of predictive classifiers. (a) Overall prediction accuracy for both test sets using classifiers trained on AFX (AFX to AGL). (b) Overall prediction accuracy for both test sets using classifiers trained on AGL (AGL to AFX). In (a) and (b), blue, yellow and brown bars represent ‘SeqMap’, ‘RefSeq’, and ‘Unigene’ for AFX, while corresponding circles faced green are for AGL. (c) Prediction accuracy for samples in each subclass using FKNN in the transfer of AFX to AGL. (d) Prediction accuracy for samples in each subclass using FKNN in the transfer of AGL to AFX.

**Figure 4(a–b)** depicts the overall prediction accuracy for both test sets where predictive classifiers generated on one platform were used to predict datasets profiled with the other platform. **Figure 4a** shows that predictive models trained with AFX data have similar predictive performance when applied to both AFX and AGL data; **Figure 4b** shows the same for models trained with AGL data, with the exception of AC 1. Combined with the corresponding T-index scores around 0.78 (**Table S3**), the results suggest that the transferability of predictive classifiers with respect to the overall performance was acceptable, except for the transfer of AFX to AGL using AC 1. Based on the previous observation of the effect of the additional 100 control samples on the transferability between AFX and AGL, we decided to conduct another analysis using the 318 common samples. The resulting decreased difference between the predictive accuracy for the AFX and AGL test sets (**Figure S5**) further confirmed the acceptable transferability of predictive classifiers. Moreover, further analysis combining data sets from AFX and AGL platforms confirmed that the classifiers trained by the combined data sets performed well for independent data sets from both AFX and AGL platforms (**Table S4**). Generally, probe-mapping methods and classification algorithms did not evidently impact on either the overall model performance or the transferability between different platforms.

The predictive performance for the individual sub-classes, however, shows a much different pattern. As shown in **Figure 4(c–d)** and **Figure S6** in which different classification algorithms were utilized, predictive models trained with AGL data show similar performance when applied to the AGL or AFX test set. Models built with the AFX data show greatly reduced predictive accuracy in the AGL test set as compared to the AFX test set, particularly for samples with scores of 1 or 2. This finding was further verified by T-index scores around or smaller than 0.5 for many cases in **Table S5**. This performance deficit appears to be consistent across both probe-mapping methods and classification algorithms**.**

We found that predictive classifiers trained on one platform could predict datasets profiled using another platform with acceptable overall predictive performance, despite slight differences between different directionality of transfer and analysis configurations (ACs). However, when the transferability was considered for each subclass, the performance of the test set that corresponded to the data used to train the model was noticeably better. Generally, the transferability of AFX to AGL was relatively poor (especially for samples with RHI scores of 1 or 2), while the transferability of AGL to AFX was much better. As was observed consistently in this study, probe-mapping methods and classification algorithms did not impact significantly on either model performance or the overall transferability.

The diversity of microarray platforms has made it a challenge to re-use and/or integrate datasets generated in different experiments to construct array-based diagnostic models. Thus, in this study, we investigated the consistency of multiple-class prediction models generated using datasets from different platforms in three aspects: the comparability of model performance from different platforms, whether predictive signature genes selected from one platform could be directly utilized to train another model on the other platform, and whether classifiers trained from one platform could predict datasets profiled from the other platform with comparable performance. The results supported the potential applications in biological and medical decision-making for cross-platform analyses of both new and existing microarray datasets. Moreover, probe-mapping methods and classification algorithms did not exert an apparent affect on either model performance or consistency between microarray platforms. However, the relatively high concordance achieved in this benchmark investigation is only the beginning of exploring cross-platform consistency because it is based on two microarray datasets generated on identical biological samples using different platforms, i.e., this investigation is mainly focused on a technical platform comparison. Undoubtedly, further studies are needed to confirm the feasibility of microarray-based cross-platform prediction, especially using independent datasets.

## Supporting Information

Figure S1 Three analysis configurations (ACs 1-3) used in this study.
(TIF)

Figure S2 Model performance for AFX after removing the additional 100 control samples in platform comparison using AC 1.
(TIF)

Figure S3 Transferability of predictive signature genes. (a) Prediction accuracy for samples in each subclass using LDA in the transfer of AFX to AGL. (b) Prediction accuracy for samples in each subclass using LDA in the transfer of AGL to AFX. (c) Prediction accuracy for samples in each subclass using SVM in the transfer of AFX to AGL. (d) Prediction accuracy for samples in each subclass using SVM in the transfer of AGL to AFX.
(TIF)

Figure S4 Model performance in transferability analysis of predictive signature genes using AC 1 after removing the additional 100 control samples in AFX.
(TIF)

Figure S5 Overall model performance for AC 1 in transferability analysis of predictive classifiers after removing the additional 100 control samples in AFX.
(TIF)

Figure S6 Transferability of predictive classifiers. (a) Prediction accuracy for samples in each subclass using LDA in the transfer of AFX to AGL. (b) Prediction accuracy for samples in each subclass using LDA in the transfer of AGL to AFX. (c) Prediction accuracy for samples in each subclass using SVM in the transfer of AFX to AGL. (d) Prediction accuracy for samples in each subclass using SVM in the transfer of AGL to AFX.
(TIF)

Table S1 Overall prediction accuracy and corresponding T-index scores for both platforms in transferability analysis of predictive signature genes.
(DOC)

Table S2 T-index scores for samples in each subclass in transferability analysis of predictive signature genes.
(DOC)

Table S3 Overall prediction accuracy and corresponding T-index scores for both platforms in transferability analysis of predictive classifiers.
(DOC)

Table S4 Prediction accuracy for models generated from the combined data.
(DOC)

Table S5 T-index scores for samples in each subclass in transferability analysis of predictive classifiers.
(DOC)

Methods S1 (DOC)

## References

[1] Fan XH, Shi LM, Fang H, Cheng YY, Perkins RG (2010). DNA microarrays are predictive of cancer prognosis: A reevaluation.. Clin Cancer Res.

[2] Gallagher WM, Tweats D, Koenig J (2009). Omic profiling for drug safety assessment: current trends and public-private partnerships.. Drug Discov Today.

[3] Gresham D, Dunham MJ, Botstein D (2008). Comparing whole genomes using DNA microarrays.. Nat Rev Genet.

[4] Pollack JR (2007). A perspective on DNA microarrays in pathology research and practice.. Am J Pathol.

[5] Shi LM, Perkins RG, Fang H, Tong WD (2008). Reproducible and reliable microarray results through quality control: good laboratory proficiency and appropriate data analysis practices are essential.. Curr Opin Biotechnol.

[6] Yauk CL, Berndt ML (2007). Review of the literature examining the correlation among DNA microarray technologies.. Environ Mol Mutagen.

[7] Hughes TR, Mao M, Jones AR, Burchard J, Marton MJ (2001). Expression profiling using microarrays fabricated by an ink-jet oligonucleotide synthesizer.. Nat Biotechnol.

[8] Kane MD, Jatkoe TA, Stumpf CR, Lu J, Thomas JD (2000). Assessment of the sensitivity and specificity of oligonucleotide (50mer) microarrays.. Nucleic Acids Res.

[9] Yuen T, Wurmbach E, Pfeffer RL, Ebersole BJ, Sealfon SC (2002). Accuracy and calibration of commercial oligonucleotide and custom cDNA microarrays.. Nucleic Acids Res.

[10] Kothapalli R, Yoder SJ, Mane S, Loughran TP (2002). Microarray results: how accurate are they?. BMC Bioinformatics.

[11] Kuo WP, Jenssen TK, Butte AJ, Ohno-Machado L, Kohane IS (2002). Analysis of matched mRNA measurements from two different microarray technologies.. Bioinformatics.

[12] Tan PK, Downey TJ, Spitznagel EL, Xu P, Fu D (2003). Evaluation of gene expression measurements from commercial microarray platforms.. Nucleic Acids Res.

[13] Shi LM, Tong WD, Fang H, Scherf U, Han J (2005). Cross-platform comparability of microarray technology: Intra-platform consistency and appropriate data analysis procedures are essential.. BMC Bioinformatics.

[14] Guo L, Lobenhofer EK, Wang C, Shippy R, Harris SC (2006). Rat toxicogenomic study reveals analytical consistency across microarray platforms.. Nat Biotechnol.

[15] Mao SH, Wang C, Dong GZ (2009). Evaluation of inter-laboratory and cross-platform concordance of DNA microarrays through discriminating genes and classifier transferability.. J Bioinform Comput Biol.

[16] Shi LM, Reid LH, Jones WD, Shippy R, Warrington JA (2006). The MicroArray Quality Control (MAQC) project shows inter- and intraplatform reproducibility of gene expression measurements.. Nat Biotechnol.

[17] Simon R, Radmacher MD, Dobbin K, McShane LM (2003). Pitfalls in the use of DNA microarray data for diagnostic and prognostic classification.. J Natl Cancer Inst.

[18] Lin YH, Friederichs J, Black MA, Mages J, Rosenberg R (2007). Multiple gene expression classifiers from different array platforms predict poor prognosis of colorectal cancer.. Clin Cancer Res.

[19] Fan XH, Lobenhofer EK, Chen MJ, Shi WW, Huang JP (2010). Consistency of Predictive Signature Genes and Classifiers Generated Using Different Microarray Platforms.. Pharmacogenomics J.

[20] Dyrskjot L, Thykjaer T, Kruhoffer M, Jensen JL, Marcussen N (2003). Identifying distinct classes of bladder carcinoma using microarrays.. Nat Genet.

[21] Sorlie T, Perou CM, Tibshirani R, Aas T, Geisler S (2001). Gene expression patterns of breast carcinomas distinguish tumor subclasses with clinical implications.. Proc Natl Acad Sci U S A.

[22] Steiner G, Suter L, Boess F, Gasser R, de Vera MC (2004). Discriminating different classes of toxicants by transcript profiling.. Environ Health Perspect.

[23] Huang L, Heinloth AN, Zeng ZB, Paules RS, Bushel PR (2008). Genes related to apoptosis predict necrosis of the liver as a phenotype observed in rats exposed to a compendium of hepatotoxicants.. BMC Genomics.

[24] Keller JM, Gray MR, Givens JA (1985). A fuzzy k-nearest neighbor algorithm.. IEEE Trans Syst Man Cybern C Appl.

[25] Shen HB, Yang J, Chou KC (2006). Fuzzy KNN for predicting membrane protein types from pseudo-amino acid composition.. J Theor Biol.

[26] Richard OD, Peter EH, David GS (2000). Pattern classification..

[27] Chang C-C, Lin C-J (2001). LIBSVM: a library for support vector machines.. http://www.csie.ntu.edu.tw/~cjlin/libsvm.

[28] Lobenhofer EK, Auman JT, Blackshear PE, Boorman GA, Bushel PR (2008). Gene expression response in target organ and whole blood varies as a function of target organ injury phenotype.. Genome Biol.

[29] Lobenhofer EK, Boorman GA, Phillips KL, Heinloth AN, Malarkey DE (2006). Application of visualization tools to the analysis of histopathological data enhances biological insight and interpretation.. Toxicol Pathol.

[30] Biganzoli E, Lama N, Ambrogi F, Antolini L, Boracchi P (2005). Prediction of cancer outcome with microarrays.. Lancet.

