# Supplementary figures and images for: Cross-Platform Comparison of Microarray-Based Multiple-Class Prediction
Source: PLoS One. 2011 Jan 11;6(1):e16067. doi: 10.1371/journal.pone.0016067 (PMC3019174; doi:10.1371/journal.pone.0016067)

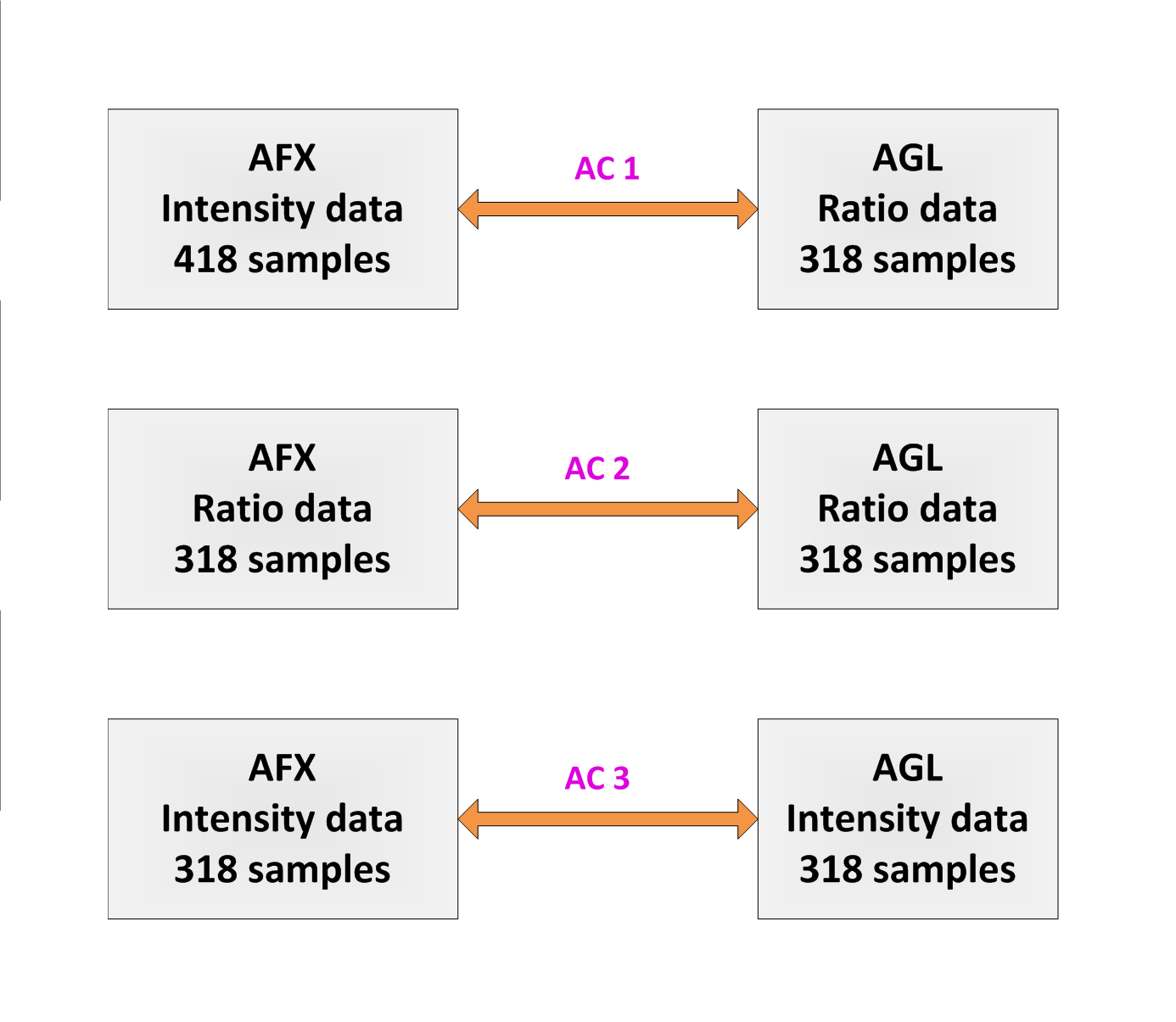

Supplement: Figure S1 — Three analysis configurations (ACs 1-3) used in this study. (TIF) [file pone.0016067.s001.tif]

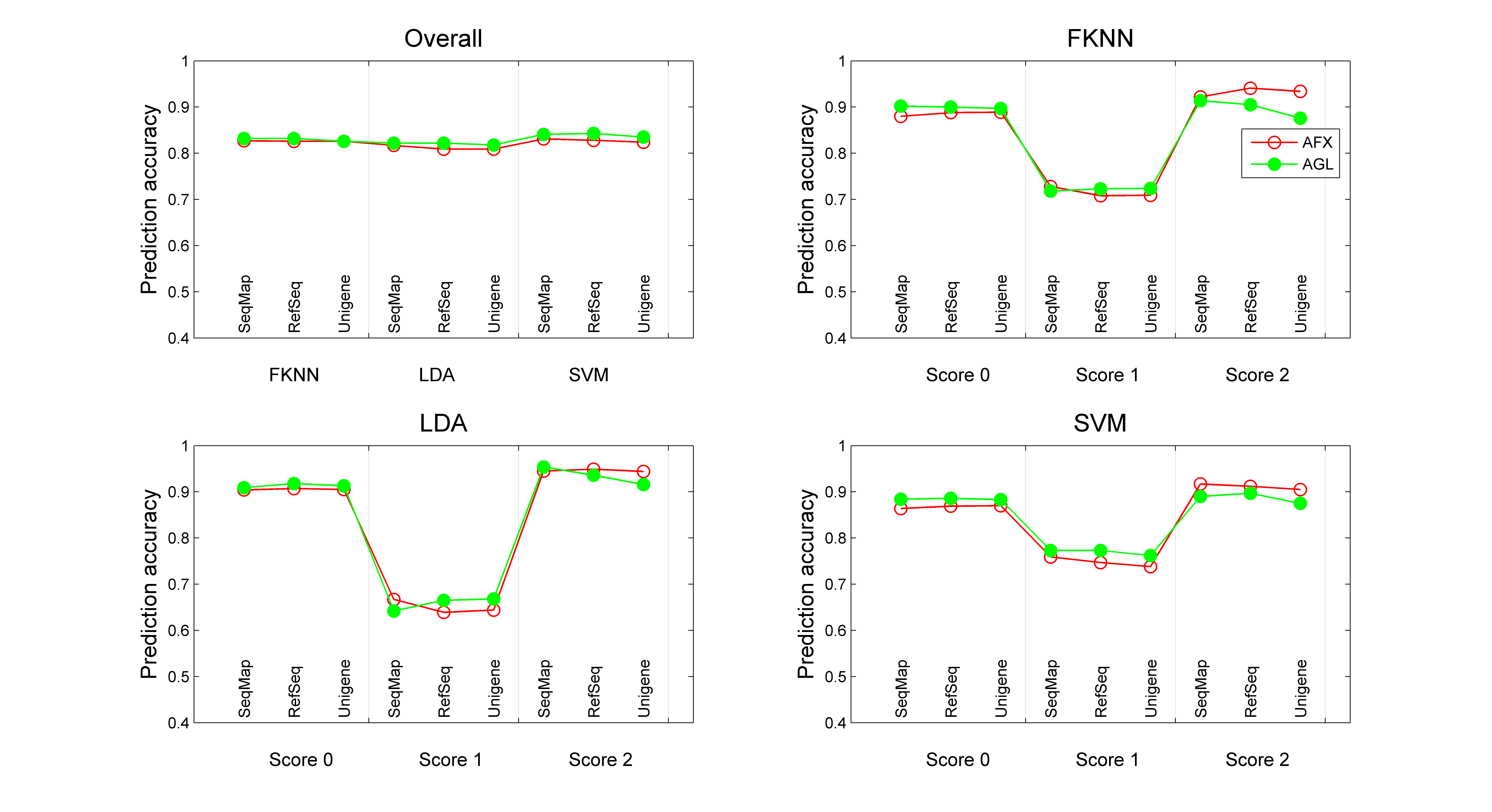

Supplement: Figure S2 — Model performance for AFX after removing the additional 100 control samples in platform comparison using AC 1. (TIF) [file pone.0016067.s002.tif]

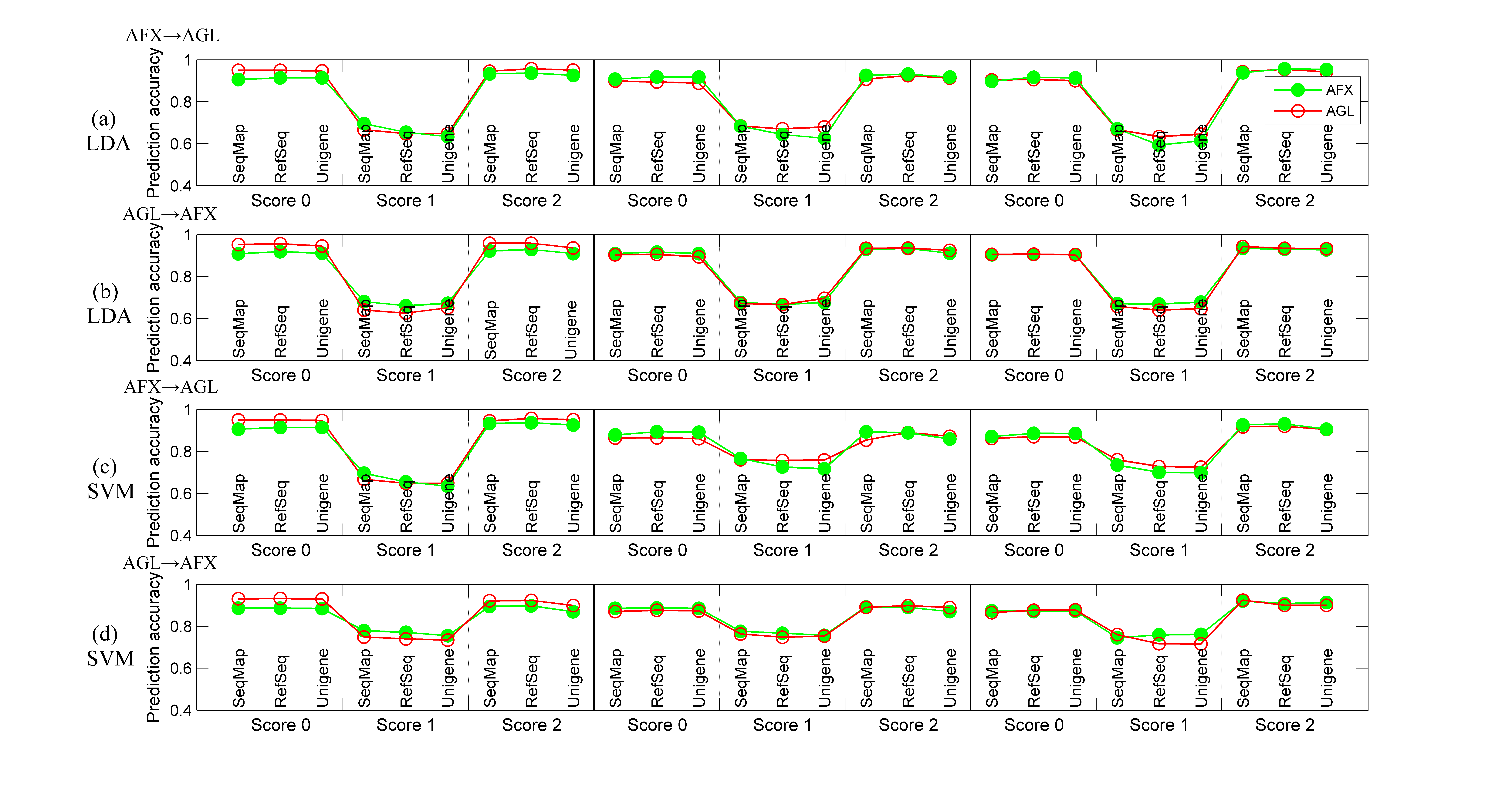

Supplement: Figure S3 — Transferability of predictive signature genes. (a) Prediction accuracy for samples in each subclass using LDA in the transfer of AFX to AGL. (b) Prediction accuracy for samples in each subclass using LDA in the transfer of AGL to AFX. (c) Prediction accuracy for samples in each subclass using SVM in the transfer of AFX to AGL. (d) Prediction accuracy for samples in each subclass using SVM in the transfer of AGL to AFX. (TIF) [file pone.0016067.s003.tif]

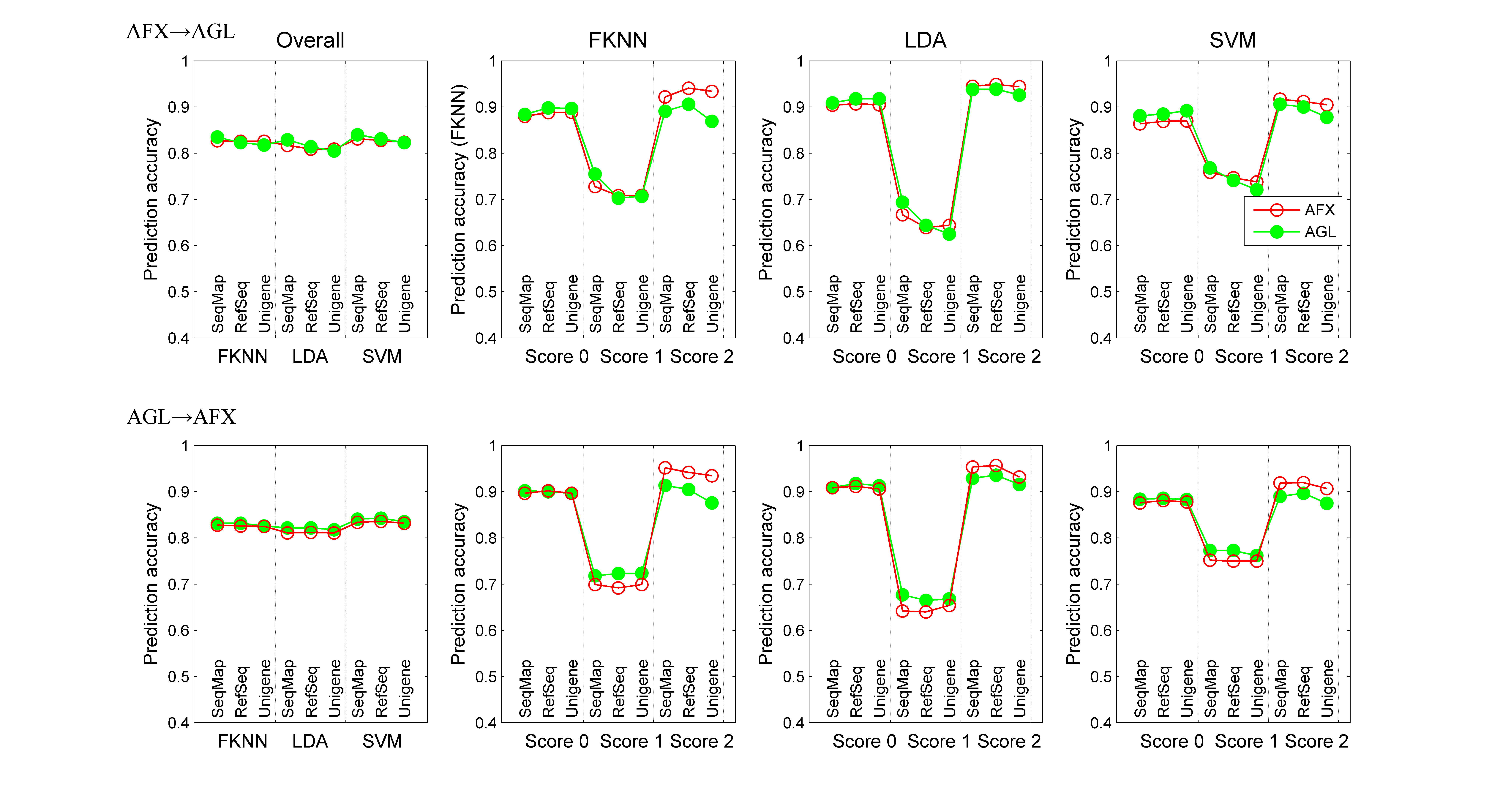

Supplement: Figure S4 — Model performance in transferability analysis of predictive signature genes using AC 1 after removing the additional 100 control samples in AFX. (TIF) [file pone.0016067.s004.tif]

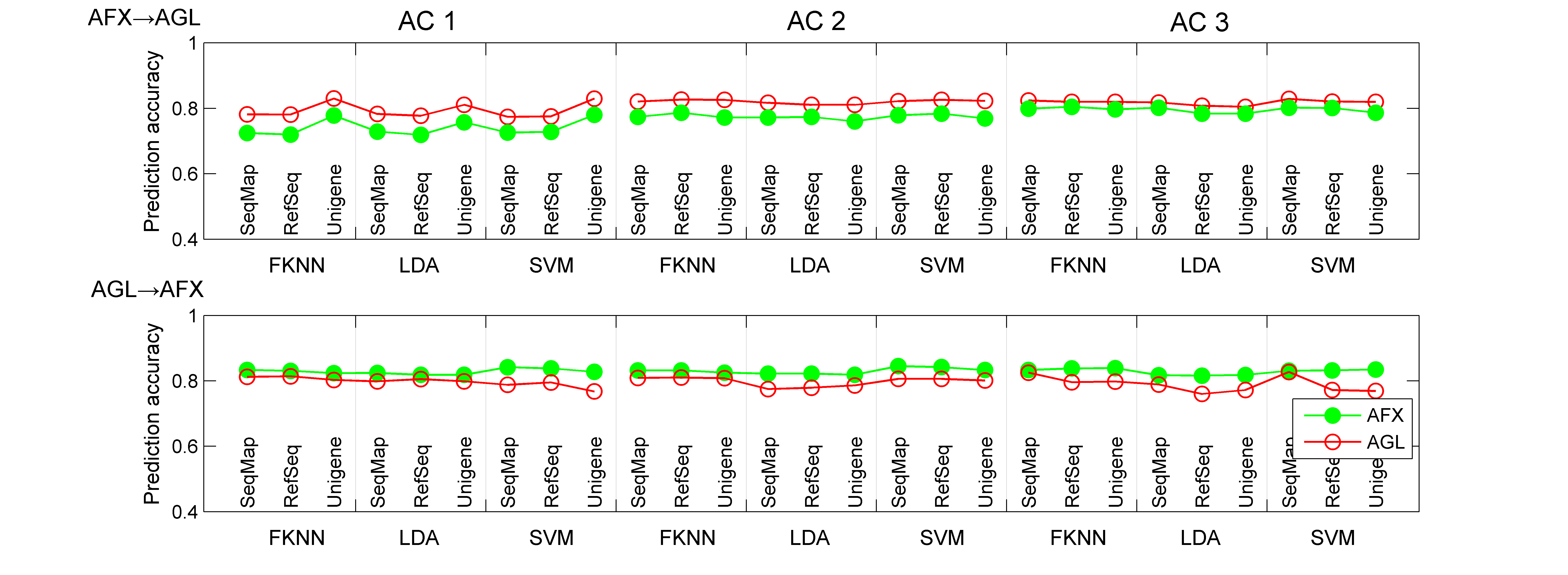

Supplement: Figure S5 — Overall model performance for AC 1 in transferability analysis of predictive classifiers after removing the additional 100 control samples in AFX. (TIF) [file pone.0016067.s005.tif]

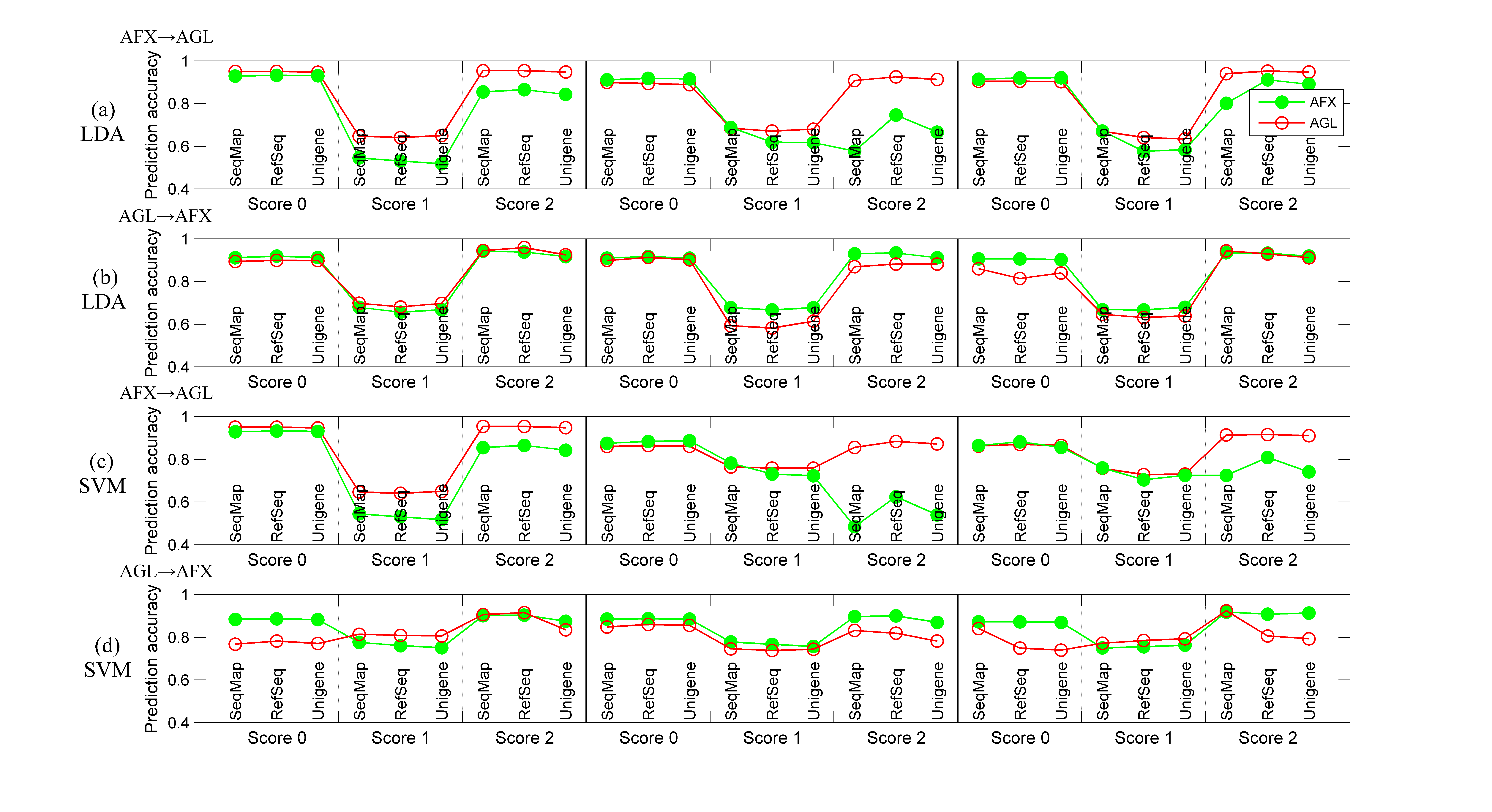

Supplement: Figure S6 — Transferability of predictive classifiers. (a) Prediction accuracy for samples in each subclass using LDA in the transfer of AFX to AGL. (b) Prediction accuracy for samples in each subclass using LDA in the transfer of AGL to AFX. (c) Prediction accuracy for samples in each subclass using SVM in the transfer of AFX to AGL. (d) Prediction accuracy for samples in each subclass using SVM in the transfer of AGL to AFX. (TIF) [file pone.0016067.s006.tif]
