# Supplementary material for: Cross-Platform Comparison of Microarray-Based Multiple-Class Prediction
Source: PLoS One. 2011 Jan 11;6(1):e16067. doi: 10.1371/journal.pone.0016067 (PMC3019174; doi:10.1371/journal.pone.0016067)
Supplement: Table S1 — Overall prediction accuracy and corresponding T-index scores for both platforms in transferability analysis of predictive signature genes. (DOC) [file pone.0016067.s007.doc]

**Table S1.** Overall prediction accuracy and corresponding T-index scores for both platforms in transferability analysis of predictive signature genes

| **Transfer** | **ACs^*^** | **Classifier** | **Common Transcript Set** | | |  |  |  |
| --- | --- | --- | --- | --- | --- | --- | --- | --- |
|  |  |  | **SeqMap** |  | **RefSeq** |  | **Unigene** |  |
|  |  |  | Accuracy | T-index | Accuracy | T-index | Accuracy | T-index |
| **AFX→AGL** | AC 1 | FKNN | 0.874→0.836 | 0.840 | 0.871→0.823 | 0.828 | 0.872→0.817 | 0.822 |
|  |  | LDA | 0.872→0.828 | 0.833 | 0.868→0.816 | 0.822 | 0.865→0.806 | 0.813 |
|  |  | SVM | 0.877→0.840 | 0.844 | 0.875→0.830 | 0.835 | 0.873→0.824 | 0.829 |
|  | AC 2 | FKNN | 0.821→0.830 | 0.829 | 0.827→0.819 | 0.820 | 0.826→0.811 | 0.813 |
|  |  | LDA | 0.817→0.823 | 0.822 | 0.811→0.813 | 0.813 | 0.811→0.804 | 0.805 |
|  |  | SVM | 0.822→0.837 | 0.835 | 0.826→0.827 | 0.827 | 0.823→0.818 | 0.819 |
|  | AC 3 | FKNN | 0.825→0.832 | 0.831 | 0.822→0.830 | 0.829 | 0.819→0.829 | 0.828 |
|  |  | LDA | 0.817→0.815 | 0.815 | 0.807→0.797 | 0.798 | 0.808→0.803 | 0.804 |
|  |  | SVM | 0.830→0.826 | 0.827 | 0.822→0.820 | 0.820 | 0.818→0.815 | 0.815 |
| **AGL→AFX** | AC 1 | FKNN | 0.834→0.872 | 0.867 | 0.831→0.869 | 0.863 | 0.826→0.869 | 0.863 |
|  |  | LDA | 0.822→0.868 | 0.861 | 0.820→0.865 | 0.858 | 0.818→0.864 | 0.857 |
|  |  | SVM | 0.845→0.880 | 0.875 | 0.843→0.878 | 0.873 | 0.831→0.873 | 0.867 |
|  | AC 2 | FKNN | 0.832→0.831 | 0.831 | 0.832→0.831 | 0.832 | 0.825→0.832 | 0.831 |
|  |  | LDA | 0.822→0.818 | 0.818 | 0.822→0.817 | 0.818 | 0.819→0.821 | 0.821 |
|  |  | SVM | 0.843→0.831 | 0.833 | 0.842→0.829 | 0.831 | 0.833→0.829 | 0.829 |
|  | AC 3 | FKNN | 0.832→0.825 | 0.826 | 0.839→0.815 | 0.818 | 0.836→0.816 | 0.819 |
|  |  | LDA | 0.817→0.815 | 0.815 | 0.817→0.807 | 0.808 | 0.819→0.808 | 0.810 |
|  |  | SVM | 0.830→0.833 | 0.833 | 0.832→0.818 | 0.820 | 0.835→0.818 | 0.820 |

^*^ ACs means analysis configurations
