# Supplementary material for: Cross-Platform Comparison of Microarray-Based Multiple-Class Prediction
Source: PLoS One. 2011 Jan 11;6(1):e16067. doi: 10.1371/journal.pone.0016067 (PMC3019174; doi:10.1371/journal.pone.0016067)
Supplement: Table S2 — T-index scores for samples in each subclass in transferability analysis of predictive signature genes. (DOC) [file pone.0016067.s008.doc]

**Table S2.** T-index scores for samples in each subclass in transferability analysis of predictive signature genes

| **Transfer** | **ACs^*^** | **Classifier** | **Common Transcript Set** | | |  |  |  |  |  |  |
| --- | --- | --- | --- | --- | --- | --- | --- | --- | --- | --- | --- |
|  |  |  | **SeqMap** |  |  | **RefSeq** |  |  | **Unigene** |  |  |
|  |  |  | Score 0 | Score 1 | Score 2 | Score 0 | Score 1 | Score 2 | Score 0 | Score 1 | Score 2 |
|  |  |  | T-index | T-index | T-index | T-index | T-index | T-index | T-index | T-index | T-index |
| **AFX→AGL** | AC 1 | FKNN | 0.886 | 0.752 | 0.890 | 0.902 | 0.701 | 0.905 | 0.898 | 0.705 | 0.861 |
|  |  | LDA | 0.907 | 0.687 | 0.933 | 0.915 | 0.653 | 0.937 | 0.915 | 0.638 | 0.926 |
|  |  | SVM | 0.882 | 0.764 | 0.909 | 0.892 | 0.734 | 0.905 | 0.892 | 0.723 | 0.892 |
|  | AC 2 | FKNN | 0.882 | 0.747 | 0.882 | 0.892 | 0.710 | 0.889 | 0.891 | 0.708 | 0.849 |
|  |  | LDA | 0.907 | 0.684 | 0.926 | 0.917 | 0.652 | 0.932 | 0.915 | 0.641 | 0.918 |
|  |  | SVM | 0.877 | 0.766 | 0.890 | 0.891 | 0.732 | 0.889 | 0.889 | 0.726 | 0.859 |
|  | AC 3 | FKNN | 0.867 | 0.742 | 0.948 | 0.894 | 0.705 | 0.952 | 0.889 | 0.711 | 0.943 |
|  |  | LDA | 0.898 | 0.669 | 0.938 | 0.916 | 0.607 | 0.957 | 0.913 | 0.624 | 0.953 |
|  |  | SVM | 0.870 | 0.740 | 0.927 | 0.884 | 0.706 | 0.931 | 0.883 | 0.704 | 0.906 |
| **AGL→AFX** | AC 1 | FKNN | 0.938 | 0.695 | 0.950 | 0.939 | 0.684 | 0.945 | 0.939 | 0.690 | 0.931 |
|  |  | LDA | 0.950 | 0.652 | 0.958 | 0.953 | 0.636 | 0.958 | 0.944 | 0.657 | 0.936 |
|  |  | SVM | 0.927 | 0.754 | 0.920 | 0.928 | 0.746 | 0.922 | 0.926 | 0.738 | 0.897 |
|  | AC 2 | FKNN | 0.890 | 0.725 | 0.922 | 0.893 | 0.724 | 0.925 | 0.891 | 0.727 | 0.919 |
|  |  | LDA | 0.904 | 0.673 | 0.935 | 0.907 | 0.666 | 0.936 | 0.895 | 0.691 | 0.924 |
|  |  | SVM | 0.871 | 0.765 | 0.890 | 0.877 | 0.752 | 0.898 | 0.874 | 0.754 | 0.888 |
|  | AC 3 | FKNN | 0.891 | 0.707 | 0.941 | 0.899 | 0.686 | 0.930 | 0.895 | 0.691 | 0.931 |
|  |  | LDA | 0.906 | 0.661 | 0.943 | 0.907 | 0.648 | 0.935 | 0.904 | 0.657 | 0.933 |
|  |  | SVM | 0.866 | 0.757 | 0.923 | 0.875 | 0.725 | 0.901 | 0.877 | 0.725 | 0.900 |

^*^ ACs means analysis configurations
