# Supplementary material for: Cross-Platform Comparison of Microarray-Based Multiple-Class Prediction
Source: PLoS One. 2011 Jan 11;6(1):e16067. doi: 10.1371/journal.pone.0016067 (PMC3019174; doi:10.1371/journal.pone.0016067)
Supplement: Table S3 — Overall prediction accuracy and corresponding T-index scores for both platforms in transferability analysis of predictive classifiers. (DOC) [file pone.0016067.s009.doc]

**Table S3.** Overall prediction accuracy and corresponding T-index scores for both platforms in transferability analysis of predictive classifiers

| **Transfer** | **ACs^*^** | **Classifier** | **Common Transcript Set** | |  |  |  |  |
| --- | --- | --- | --- | --- | --- | --- | --- | --- |
|  |  |  | **SeqMap** |  | **RefSeq** |  | **Unigene** |  |
|  |  |  | Accuracy | T-index | Accuracy | T-index | Accuracy | T-index |
| **AFX→AGL** | AC 1 | FKNN | 0.873→0.783 | 0.792 | 0.874→0.778 | 0.788 | 0.875→0.770 | 0.780 |
|  |  | LDA | 0.867→0.767 | 0.778 | 0.866→0.765 | 0.775 | 0.866→0.756 | 0.767 |
|  |  | SVM | 0.876→0.788 | 0.796 | 0.876→0.792 | 0.800 | 0.876→0.781 | 0.790 |
|  | AC 2 | FKNN | 0.821→0.774 | 0.780 | 0.827→0.787 | 0.792 | 0.826→0.772 | 0.779 |
|  |  | LDA | 0.817→0.772 | 0.778 | 0.811→0.774 | 0.780 | 0.811→0.760 | 0.768 |
|  |  | SVM | 0.822→0.779 | 0.784 | 0.826→0.784 | 0.790 | 0.823→0.769 | 0.776 |
|  | AC 3 | FKNN | 0.824→0.799 | 0.803 | 0.820→0.805 | 0.808 | 0.820→0.797 | 0.800 |
|  |  | LDA | 0.818→0.802 | 0.804 | 0.808→0.784 | 0.788 | 0.805→0.784 | 0.788 |
|  |  | SVM | 0.829→0.802 | 0.806 | 0.821→0.801 | 0.804 | 0.820→0.787 | 0.792 |
| **AGL→AFX** | AC 1 | FKNN | 0.836→0.840 | 0.840 | 0.831→0.841 | 0.839 | 0.825→0.826 | 0.826 |
|  |  | LDA | 0.824→0.845 | 0.842 | 0.819→0.846 | 0.842 | 0.817→0.846 | 0.841 |
|  |  | SVM | 0.844→0.796 | 0.800 | 0.840→0.804 | 0.808 | 0.830→0.788 | 0.793 |
|  | AC 2 | FKNN | 0.832→0.809 | 0.812 | 0.832→0.810 | 0.813 | 0.825→0.808 | 0.811 |
|  |  | LDA | 0.822→0.775 | 0.781 | 0.822→0.779 | 0.785 | 0.819→0.786 | 0.791 |
|  |  | SVM | 0.845→0.806 | 0.811 | 0.842→0.806 | 0.811 | 0.833→0.801 | 0.805 |
|  | AC 3 | FKNN | 0.833→0.825 | 0.826 | 0.838→0.796 | 0.800 | 0.839→0.798 | 0.803 |
|  |  | LDA | 0.817→0.789 | 0.793 | 0.816→0.760 | 0.767 | 0.818→0.772 | 0.778 |
|  |  | SVM | 0.831→0.827 | 0.827 | 0.832→0.772 | 0.778 | 0.835→0.769 | 0.775 |

* ACs means analysis configurations
