# Supplementary material for: Cross-Platform Comparison of Microarray-Based Multiple-Class Prediction
Source: PLoS One. 2011 Jan 11;6(1):e16067. doi: 10.1371/journal.pone.0016067 (PMC3019174; doi:10.1371/journal.pone.0016067)
Supplement: Table S4 — Prediction accuracy for models generated from the combined data. (DOC) [file pone.0016067.s010.doc]

**Table S4**. Prediction accuracy for models generated from the combined data

|  |  | **Training set^*^** | **Validation (AFX)**** | **Validation (AGL) **** |
| --- | --- | --- | --- | --- |
| **AC 1** | RefSeq | 0.905 | 0.838 | 0.785 |
|  | SeqMap | 0.903 | 0.840 | 0.786 |
|  | Unigene | 0.902 | 0.836 | 0.776 |
| **AC 2** | RefSeq | 0.915 | 0.779 | 0.785 |
|  | SeqMap | 0.913 | 0.780 | 0.788 |
|  | Unigene | 0.913 | 0.776 | 0.783 |
| **AC 3** | RefSeq | 0.900 | 0.778 | 0.785 |
|  | SeqMap | 0.900 | 0.782 | 0.785 |
|  | Unigene | 0.901 | 0.777 | 0.784 |

^*^Leave-one-out cross-validation results.

^**^The models are trained by the combined training sets and challenged by corresponding test set.
