# Supplementary material for: Cross-Platform Comparison of Microarray-Based Multiple-Class Prediction
Source: PLoS One. 2011 Jan 11;6(1):e16067. doi: 10.1371/journal.pone.0016067 (PMC3019174; doi:10.1371/journal.pone.0016067)
Supplement: Table S5 — T-index scores for samples in each subclass in transferability analysis of predictive classifiers. (DOC) [file pone.0016067.s011.doc]

**Table S5.** T-index scores for samples in each subclass in transferability analysis of predictive classifiers

| **Transfer** | **ACs^*^** | **Classifier** | **Common Transcript Set** | |  |  |  |  |  |  |  |
| --- | --- | --- | --- | --- | --- | --- | --- | --- | --- | --- | --- |
|  |  |  | **SeqMap** |  |  | **RefSeq** |  |  | **Unigene** |  |  |
|  |  |  | Score 0 | Score 1 | Score 2 | Score 0 | Score 1 | Score 2 | Score 0 | Score 1 | Score 2 |
|  |  |  | T-index | T-index | T-index | T-index | T-index | T-index | T-index | T-index | T-index |
| **AFX→AGL** | AC 1 | FKNN | 0.922 | 0.633 | 0.809 | 0.924 | 0.618 | 0.825 | 0.926 | 0.609 | 0.791 |
|  |  | LDA | 0.930 | 0.576 | 0.851 | 0.933 | 0.565 | 0.861 | 0.931 | 0.558 | 0.838 |
|  |  | SVM | 0.910 | 0.674 | 0.753 | 0.915 | 0.669 | 0.789 | 0.912 | 0.666 | 0.727 |
|  | AC 2 | FKNN | 0.883 | 0.738 | 0.482 | 0.890 | 0.701 | 0.685 | 0.891 | 0.687 | 0.602 |
|  |  | LDA | 0.910 | 0.687 | 0.501 | 0.916 | 0.632 | 0.710 | 0.914 | 0.632 | 0.615 |
|  |  | SVM | 0.873 | 0.779 | 0.428 | 0.882 | 0.736 | 0.585 | 0.884 | 0.729 | 0.480 |
|  | AC 3 | FKNN | 0.879 | 0.729 | 0.714 | 0.897 | 0.681 | 0.860 | 0.877 | 0.695 | 0.812 |
|  |  | LDA | 0.913 | 0.671 | 0.784 | 0.919 | 0.596 | 0.910 | 0.920 | 0.600 | 0.887 |
|  |  | SVM | 0.864 | 0.760 | 0.704 | 0.881 | 0.710 | 0.803 | 0.857 | 0.726 | 0.721 |
| **AGL→AFX** | AC 1 | FKNN | 0.860 | 0.748 | 0.956 | 0.862 | 0.752 | 0.956 | 0.842 | 0.749 | 0.917 |
|  |  | LDA | 0.894 | 0.693 | 0.945 | 0.900 | 0.674 | 0.960 | 0.898 | 0.689 | 0.925 |
|  |  | SVM | 0.772 | 0.807 | 0.906 | 0.787 | 0.800 | 0.915 | 0.775 | 0.795 | 0.834 |
|  | AC 2 | FKNN | 0.907 | 0.680 | 0.875 | 0.904 | 0.686 | 0.874 | 0.902 | 0.692 | 0.850 |
|  |  | LDA | 0.900 | 0.614 | 0.865 | 0.913 | 0.604 | 0.879 | 0.902 | 0.630 | 0.881 |
|  |  | SVM | 0.851 | 0.752 | 0.831 | 0.862 | 0.743 | 0.818 | 0.858 | 0.747 | 0.781 |
|  | AC 3 | FKNN | 0.881 | 0.715 | 0.947 | 0.822 | 0.732 | 0.879 | 0.827 | 0.736 | 0.875 |
|  |  | LDA | 0.862 | 0.653 | 0.944 | 0.812 | 0.641 | 0.929 | 0.841 | 0.650 | 0.911 |
|  |  | SVM | 0.844 | 0.767 | 0.924 | 0.750 | 0.779 | 0.798 | 0.739 | 0.788 | 0.782 |

* ACs means analysis configurations
