# Supplementary material for: Cross-Platform Comparison of Microarray-Based Multiple-Class Prediction
Source: PLoS One. 2011 Jan 11;6(1):e16067. doi: 10.1371/journal.pone.0016067 (PMC3019174; doi:10.1371/journal.pone.0016067)
Supplement: Methods S1 — (DOC) [file pone.0016067.s012.doc]

**Supplementary Methods**

The *best classifier* was derived from the method used in our previous study[19]. Details are described below and in the footnote Figure 1(a):

1. Stratified random sample splitting – We used 70/30 splitting, where the 70% and 30% samples were used for model construction and validation, respectively. Since the sample splitting was random in nature, the whole process was repeated 500 times, thereby generating 500 best classifiers. The results of these 500 classifiers were used to calculate T-index scores.
2. Feature filtering – This step was employed to generate an initial pool of probes used in subsequent analyses. The number of probes was around 300 after filtering with a fold-change threshold of 2 and a *p*-value criterion of 0.05. These probes were then rank-ordered based on absolute fold change values.
3. Feature selection – A sequential forward feature selection method was applied, where probes associated with the best performance were added into the model step by step. The resulting classifier was then used to predict the remaining 30% samples and the prediction accuracy was recorded (see step 4).
4. Prediction – For classifier *i* (*i* is the number of probes used in the classifier), if the subsequent five consecutive classifiers performed worse than or equal to that of classifier *i*, the procedure stopped and classifier *i* was selected as the best classifier. If the predication accuracy was greater than that of classifier *i*, steps 3 and 4 are repeated.
